# Through the Google Glass: The impact of heads-up displays on visual attention

**DOI:** 10.1186/s41235-016-0015-6

**Published:** 2016-11-05

**Authors:** Joanna E. Lewis, Mark B. Neider

**Affiliations:** Department of Psychology, University of Central Florida, 4111 Pictor Lane, Psychology Building 99, Suite 320, Orlando, FL 32816-1390 USA

**Keywords:** Visual Search, Search Task, Primary Task, Inattentional Blindness, Recognition Memory Task

## Abstract

In five experiments, we evaluated how secondary information presented on a heads-up display (HUD) impacts performance of a concurrent visual attention task. To do so, we had participants complete a primary visual search task under a variety of secondary load conditions (a single word presented on Google Glass during each search trial). Processing of secondary information was measured through a recognition memory task. Other manipulations included relevance (Experiments 1–4) and temporal onset of secondary information relative to the primary task (Experiment 3). Secondary information was always disruptive to the visual search, regardless of temporal onset and even when participants were instructed to ignore it. These patterns were evident in search tasks reflective of both selective (Experiments 1–3) and preattentive (Experiment 4) attentional mechanisms, and were not a result of onset-offset attentional capture (Experiment 5). Recognition memory for secondary information was always above chance. Our findings suggest that HUD-based visual information is profoundly disruptive to attentional processes and largely immune to user-centric prioritization.

## Significance

In five experiments, we break new empirical ground by characterizing dual-task impairments associated with secondary information presented on a heads-up display (HUD) (i.e., Google Glass) during a primary visual search task. By combining two classical cognitive psychology paradigms (visual search and recognition memory), our studies dissociate impairments to selective and preattentive mechanisms while quantifying the extent to which secondary information is processed in a context increasingly encountered in the real world (e.g., information projected on a windshield when driving). Our results indicate that secondary HUD-based information is ubiquitously disruptive to attentional mechanisms, independent of user-centric prioritization and the time course of secondary information.

## Background

Our lives are being continuously and increasingly intermingled with technology (e.g., smartphones, wearable HUDs). While creating informationally rich environments might lead to productivity benefits in some contexts and convenience in others, designers, scientists, and users need to understand how technological integration might also be harmful. We investigate this latter context in our present research, which contains a unique blend of theoretically relevant and practically applicable data that should be of interest to a wide audience, including psychologists, engineers, designers, policy makers, and the general public.

Mobile technology has become essential and pervasive in the everyday lives of many people. Understanding the extent to which increasingly integrated information systems, such as cell phones (Drews, Yazdani, Godfrey, Cooper, & Strayer, 2009; Strayer, Drews, & Johnston, 2003) and other user interfaces, impact human performance on a range of common tasks and cognitive processes is of critical importance. Specifically, how does the adoption of various technologies remove a user from the present moment or task at hand, and at what cost (Starner, 2002)? Mobile technologies, for instance, have progressed from cell phones to wearable interfaces, leaving users in constant contact with their devices, regardless of whether they explicitly choose to engage with that device.

It has been well established that engaging in multitasking induces costs to performance (Allport, 1980; Horrey & Wickens, 2006; Neider, McCarley, Crowell, Kaczmarski, & Kramer, 2010; Strayer et al., 2003). In the practical domain, much of this research is focused on cell phone engagement in the context of driving or walking (Horrey & Wickens, 2006; Kramer, Hahn, Irwin, & Theeuwes, 1999; Neider et al., 2010). For example, using a cell phone or text-to-speech interface while driving significantly increases cognitive load and crash risk (Drews et al., 2009; Strayer et al., 2013), and it impairs memory for visual information (Strayer et al., 2003). While a focus on cell phone-related distraction has made practical sense, given the approximately 7.1 billion mobile subscriptions internationally (International Telecommunication Union ITU, 2015), emergent technologies are moving toward a user-integrated approach favoring HUDs. HUDs have long been used in aviation cockpits and are now being employed in everyday environments, such as automobiles (e.g. Cadillac and Mercedes vehicles), or integrated directly with the user, such as with Google Glass (GG) and Oculus Rift (Ceurstemont, 2014). Unlike cell phones, HUDs typically present users with a persistent stream of visual information (though systems such as GG can provide auditory information as well), increasing the likelihood of interference with other concurrent visual tasks (Wickens, 2002, 2008). Although prior work in the multitasking domain is largely ubiquitous in demonstrating performance impairments under such conditions across a variety of contexts, novel reappropriations of existing technologies can carry with them some implicit expectation that they might immunize against such impairments. HUDs, which make use of transparent displays, have been used with great success in the aviation domain; however, the information-processing needs and priorities of a pilot at 30,000 feet are likely to be very different from those of a driver on the ground who might have only seconds to respond to a potential hazard. Consequently, as HUDs become increasingly used in less specialized contexts, it becomes imperative to understand how they might impact overall behavior when set against attentional limitations. To date, the literature relating HUD-based technology to attention and performance costs in everyday contexts has been minimal (Starner, 2002; Wolffsohn, McBrien, Edgar, & Stout, 1998).

Our goals in the present experiments were twofold. First, we wanted to characterize the extent to which visual information presented on a user-worn HUD (e.g., GG) impacts performance on a primary visual task, and how such effects might be modulated by the relevance and temporal presentation (i.e., onset prior to, concurrently, or following onset of primary task) of the HUD-based information. Second, we wanted to shed light on possible attentional mechanisms underlying performance costs arising from information presented on HUDs while engaged in a concurrent primary task (analogous to conversing on a cell phone while driving). To do so, we employed a visual search paradigm as our primary task, allowing us to isolate impairments to both parallel and serial attention mechanisms. Whereas efficient search for singleton targets is thought to involve parallel, preattentive processes (and less so selective attention), searches that are inefficient are thought to require serial attention processes that rely heavily on selective attention (Wolfe, 1998). Critically, if performance impairments occurred only during inefficient search, it would suggest that secondary task information presented on the GG is largely detrimental to selective attentional processes, perhaps those related to efficiently guiding attention toward the target. Alternatively, if secondary information presented on the GG induces performance costs during singleton search, it would suggest impairment to preattentive processes as well (though it would not rule out some impairment to selective attention mechanisms), and more generally to broader visual processing. An additional benefit of using a search task is that search is a vital operation for everyday function; humans must constantly locate task-relevant information (such as a pedestrian about to run into a roadway) in the environment. Thus, visual search is both a theoretically useful and practically relevant paradigm to assess HUD-based dual-task effects.

In all experiments, the participant’s primary task was to locate a T target among L distractors displayed on a computer screen. In some conditions, the secondary information, in the form of a single word, was concurrently presented on a GG that was worn during a portion of the experiment. In Experiment 1, we characterized primary task performance costs associated with the presentation of secondary information on the GG while also manipulating the perceived relevance of the secondary information (through instructions) to the participant. We predicted response time (RT) costs to the visual search task in the presence of a secondary information stream, as well as an added cost when participants were told the information was useful. The extent to which secondary task information was processed was assessed through a surprise recognition memory task administered after all search trials were completed. In Experiment 2, we manipulated the context of the secondary information presented on the GG by informing participants of the recognition memory task. We expected secondary information to be more disruptive to the primary task when participants were aware that they would be tested on it. In Experiment 3, we explored the degree to which variation in the time course of the onset of secondary information impacted primary task performance (prior to, concurrently, or following the primary task), and the extent to which this might interact with the perceived relevance of that information. We expected concurrent presentation to produce larger costs to primary task performance, with this cost increasing when the secondary task was perceived as more relevant. In Experiment 4, we manipulated the saliency of the target T to elicit singleton search behavior to evaluate whether performance costs are exclusive to selective attention mechanisms or exist for preattentative processes as well. In the final experiment, we masked the onset and offset of the secondary task information to guard against the possibility that our effects might be more closely related to some reflexive reorienting of attentional processes toward an abrupt stimulus onset, as opposed to informational processing impairments associated with managing dual-task demands.

## Experiment 1

### Methods

#### Participants

Ninety participants from the University of Central Florida’s undergraduate research pool participated (56 females, *M* age = 19.58) for course credit. Eighteen participants were assigned to each experimental condition, based on previous research (Neider et al., 2010). We controlled for noise or experimental errors by replacing any participant who was run in a noisy environment or incorrectly with another subject using the same condition. All participants had normal or corrected-to-normal visual acuity and normal color vision. Consent was obtained prior to screening and experimentation, as per the Declaration of Helsinki. This research was approved by University of Central Florida’s Institutional Review Board (IRB Number SBE-14-10257). The total experiment took about 1 h to complete.

#### Experimental tasks

##### Primary task

**Fig. 1:**
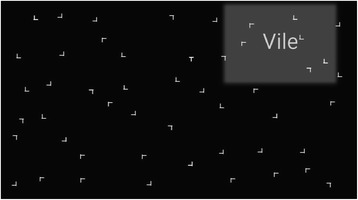
Example of the experimental environment in all experiments (target was red in Experiment 4) from the participant’s perspective

The primary task in all of the experiments was a visual search task. Participants responded to the orientation (90 degrees or −90 degrees) of a T target among L distractors. To increase difficulty of the search task, (1) we used large set sizes (50 or 80 items per trial), and (2) the intersection of the L distractors was offset by 2 pixels (search items were 20 × 20 pixels, 0.72-degree visual angle) to increase target-distractor similarity (see Fig. 1). By using a difficult search task for the primary task, we were able to reasonably ensure that the secondary information presented on the GG would appear to each participant for the same amount of time; that is, it was highly unlikely that a participant would find the T target prior to the onset, and subsequent offset, of the secondary information on the GG. The primary task timed out after 12 seconds. Participants completed 30 practice trials and then 120 search trials.

##### Secondary task 

There were five GG conditions associated with secondary task load. To create a baseline and a control for any visual occlusion that might occur when wearing the GG, we included two conditions where no secondary information was presented. These no-load conditions had participants performing the search task without wearing the GG (control) or while wearing the GG with no information presented on it (glass only). The other three GG conditions were similar, except that secondary information (a single word) was presented on the GG for 2000 milliseconds while the participant concurrently performed the search task (dual-task conditions). In conditions where secondary information was presented on the GG, participants were instructed that (1) they should ignore the information on the GG, (2) the information on the GG was irrelevant, or (3) the information on the GG might be useful for the primary task. Regardless of instruction, secondary information was never meaningful for the primary task. The words appeared simultaneously with the onset of the search display onset. The GG screen display size was approximately 2.5 degrees of visual angle. Words for the secondary task were randomly selected from the MRC Psycholinguistic Database (Coltheart, 1981), based on the parameters of length (4–7 letters), syllables (3 or fewer), and frequency in the English language (frequency range of 15–100).

##### Recognition task

A surprise recognition memory task was administered following the completion of the primary experimental task to determine the extent to which secondary words were processed in the dual-task conditions (Jones, Jacoby, & Gellis, 2001). Previously seen words and new words (60 of each), which were sampled using the aforementioned parameters, were interspersed, and participants were asked to respond whether the word was presented when they performed the main experimental task. Each word was presented for 1500 milliseconds, followed by six asterisks to cue a response. During 16 practice trials, participants received feedback regarding the accuracy of their responses. Throughout the recognition task, if the participants failed to respond during the cue display, they received feedback that they had not responded. Nonresponses were counted as errors during analysis.

#### Design and procedure

In experiment 1, we employed a mixed factorial design, with set size (50 or 80 search items) as a within-subject factor and GG conditions (control, glass, and dual-task conditions) as a between-subjects factor. Participants completed 30 practice trials of the search task without the secondary task. Following a brief break, each participant received instructions (between subjects) regarding the relevancy of the information presented on the GG. Participants in all conditions except the control were instructed to place the GG on so that the screen was visible and aligned with the top edge of the computer monitor. Following the completion of the visual search task, participants were instructed to return the GG to the researchers and read instructions regarding the word recognition memory task. They completed 16 practice trials before beginning the recognition memory task.

## Results and discussion

**Fig. 2:**
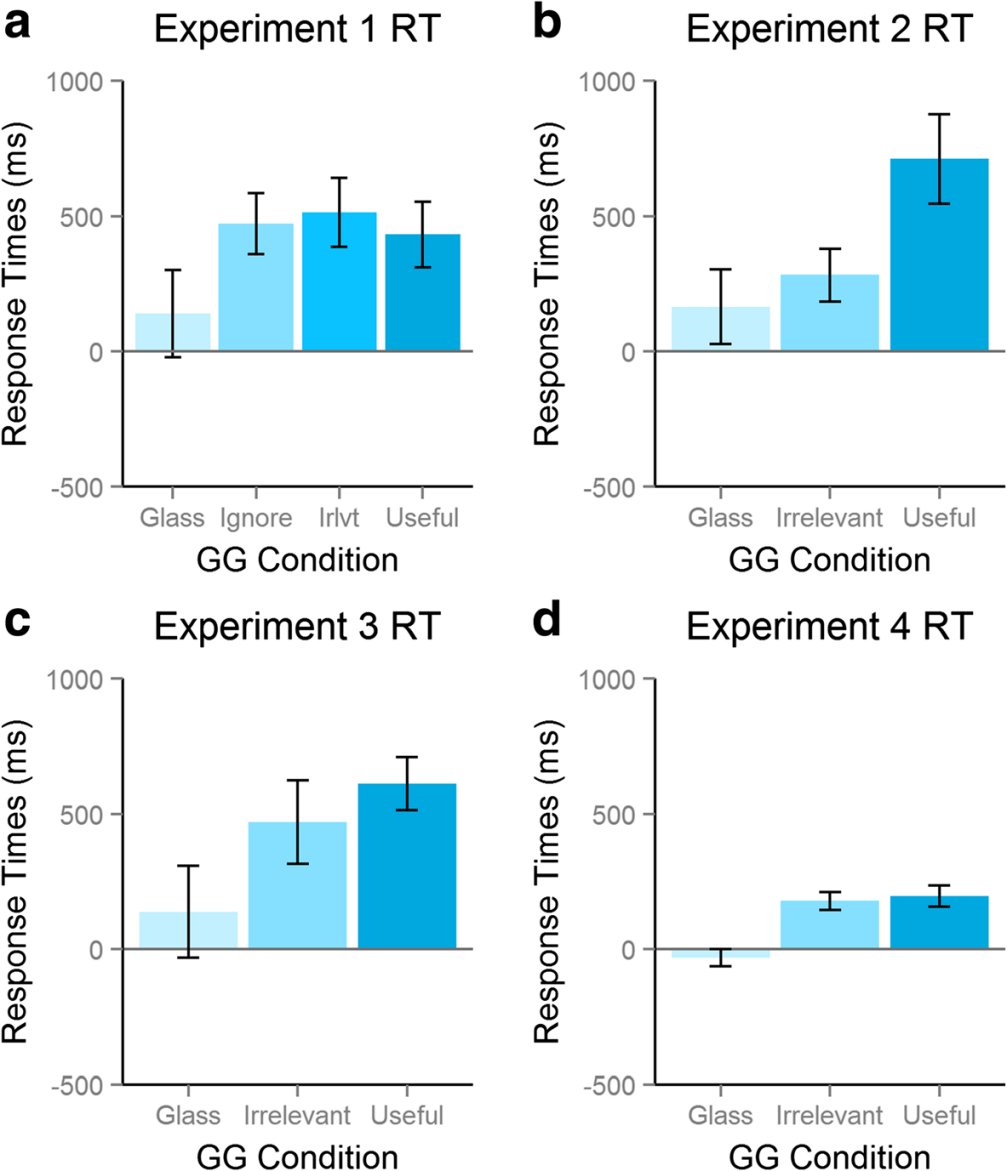
Response times (RTs) relative to control condition (Google Glass [GG] condition-control) in Experiments **a** 1, **b** 2, **c** 3, and **d** 4. Error bars indicate SEM

**Fig. 3:**
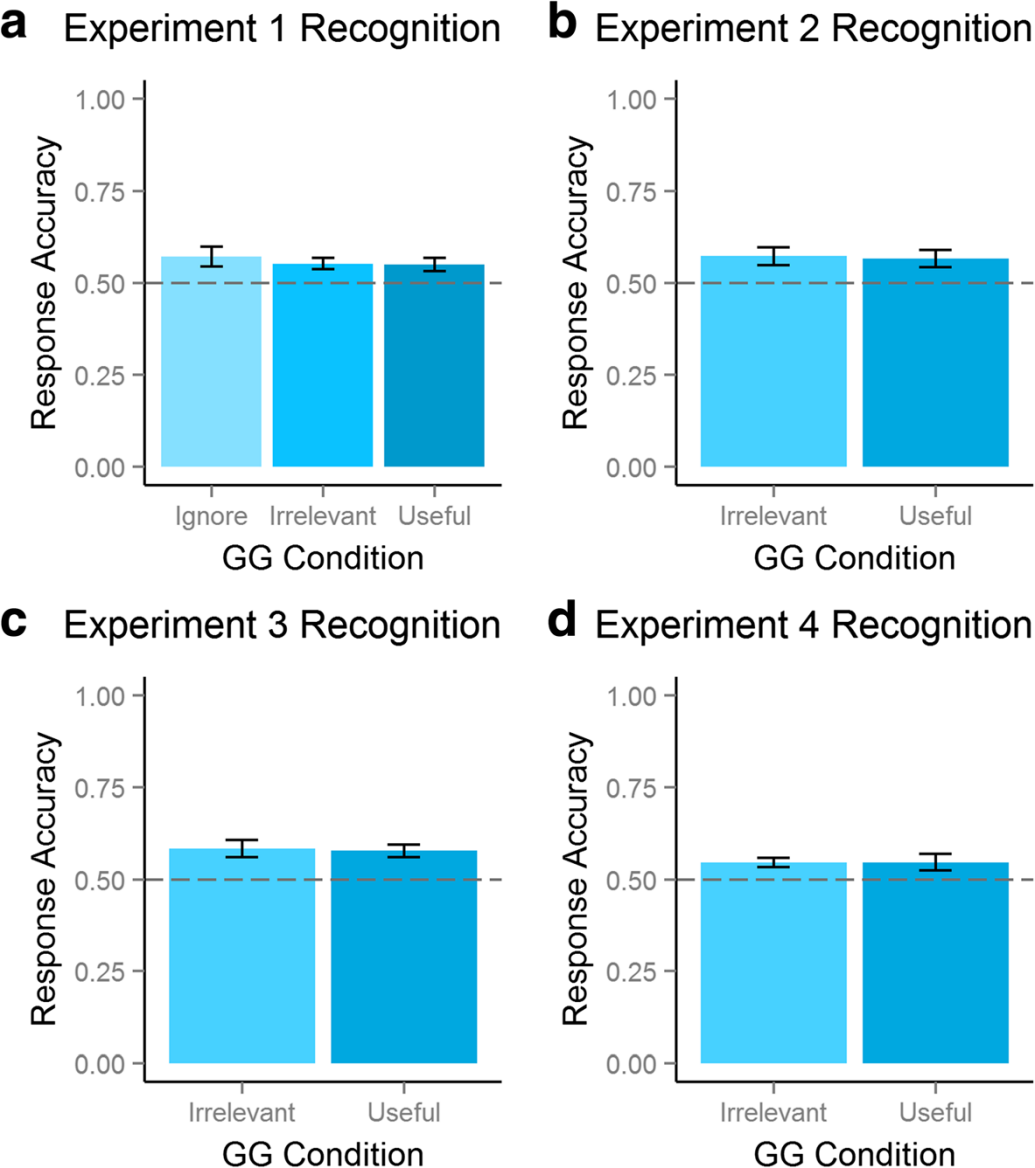
Recognition memory accuracy in Experiments **a** 1, **b** 2, **c** 3, and **d** 4 as a function of Google Glass (GG) condition. Error bars indicate SEM, and *dashed lines* indicate chance (50 %) accuracy

Eight participants were removed due to accuracy or reaction time (RT) values more than 2 SD from the mean. We found no differences for accuracy across conditions (see Table 1). RTs relative to the control (condition-control) are shown in Fig. 2; positive values indicate RT costs relative to the control condition. There was a main effect of GG condition on RT (*F*[4, 77] = 2.70, *p* = .037, η^2^ = .123), suggesting that participants took longer to perform the primary search task when secondary information was presented (see Table 2). Additionally, we found an RT cost for the dual-task conditions compared with both the control and no information presented conditions (*p*_s_ < .05). There were no differences between the dual-task conditions (*p*_s_ > .05). We also found a main effect of set size (*F*[1, 77] = 63.21, *p* < .001, η^2^ = .451), but no interaction between set size and GG condition (*F*[4, 77] = 0.46, *p* = .766, η^2^ = .023). RT × set size function slopes averaged 20.80 milliseconds per item. Accuracy in the recognition memory task was analyzed using one-sample *t* tests with participant performance compared against 50 % accuracy, which constituted chance accuracy. Memory performance was significantly above chance in the recognition task (all *p*_s_ < .05), with no differences across dual-task conditions (*F*[2, 45] = 0.49, *p* = .613, η^2^ = .021) (see Fig. 3), indicating that participants processed secondary information independent of instructions given.

**Table 1:** Visual search accuracy for Experiments 1–5

Experiment	Accuracy	*F*-Test
1	0.75 (0.09)	*F*(4, 77) = 0.65, *p* = .627
2	0.75 (0.13)	*F*(3, 61) = 2.15, *p* = .103
3	0.69 (0.12)	*F*(3, 61) = 0.57, *p* = .636
4	0.99 (0.01)	*F*(3, 58) = 1.65, *p* = .187
5	0.74 (0.15)	*F*(2, 53) = 0.04, *p* = .960

Response accuracy means and statistical significance (main effect of Google Glass condition) for the visual search task for all experiments

**Table 2:** Search response times for Experiments 1–5

Experiments	Mean RT (milliseconds)	SD
Experiment 1		
Control	5374.37	727.51
No Glass	5490.74	677.60
Ignore	5847.93	464.90
Irrelevant	5889.40	524.73
Useful	5758.65	488.22
Experiment 2		
Control	5310.86	595.89
No Glass	5476.08	567.35
Irrelevant	5594.44	376.06
Useful	6024.03	681.85
Experiment 3		
Control	5433.95	587.93
No Glass	5572.86	676.36
Irrelevant	5903.77	621.21
Useful	6047.32	390.88
Experiment 4		
Control	902.65	201.63
No Glass	871.82	126.54
Irrelevant	1082.63	126.50
Useful	1099.38	157.75
Experiment 5		
Control	5126.83	605.69
Irrelevant	5683.76	542.43
Useful	5588.50	622.03

Response time (RT) means and SDs for the visual search task for all experiments by each condition

## Experiment 2

Generally speaking, presenting information on the GG concurrently with the search task induced costs to RT performance. However, participants were unaware that they would be tested on the secondary information presented on the GG, which may have disproportionately biased them toward discounting that information. To address this possibility, in Experiment 2 we informed participants of the memory recognition test.

### Methods

Seventy-two naive participants (52 females, *M* age = 19.89) were recruited for Experiment 2. All experimental details were identical to those in Experiment 1, except for the following three changes: (1) We eliminated the dual-task ignore condition because of the similarity to the dual-task irrelevant condition; (2) we changed all of the dual-task instructions to manipulate the secondary task relationship to the entire experiment as opposed to just the visual search task; and (3) we informed the participants of the recognition memory task.

### Results

Seven participants were excluded from the analyses because of accuracy or RT values more than 2 SD from the mean. Overall, the data were similar to those in Experiment 1. We found no differences for accuracy across conditions (see Table 1). When secondary information was presented, participants took longer to perform the primary search task (*F*[3, 61] = 4.16, *p* = .010, η^2^ = .170) and dual-task conditions. RTs were significantly different from the control conditions (*p*_s_ < .05). Interestingly, search RTs were longer when purportedly useful secondary information was presented on the GG (*p* < .05) (see Fig. 2b). We also found an effect of set size (*F*[1, 61] = 71.73, *p* < .001, η^2^ = .540), but no interaction between set size and GG condition (*F*[3, 61] = 0.43, *p* = .731, η^2^ = .021). RT × set size function slopes averaged 22.23 milliseconds per item. Memory performance was significantly above chance (all *p*_s_ < .05) in the recognition task, with no difference between conditions (*F*[1, 29] = 0.00, *p* = .952, η^2^ = .004).

### Discussion

The data patterns derived from the first two experiments are both surprising and alarming. Participants were unable to filter out secondary information presented on the GG. More practically, our data strongly suggest that observers cannot completely inhibit secondary information presented on a HUD, even when they want to or are instructed to do so. Perhaps equally concerning, when participants in Experiment 2 were biased to attend to HUD-based information (i.e., instructed the information might be useful), RTs increased by about 86 %. A real-world analogue would be an individual receiving a text message or visual route information on a HUD while driving and choosing to allocate attention to this secondary information at the expense of performance on the primary task.

## Experiment 3

Our data derived from Experiments 1 and 2 clearly suggest that secondary information presented on a HUD elicits RT costs to concurrent tasks involving visual attention; however, the data are limited to cases where the task information is time-locked to onset concurrently. In the real world, information ebbs and flows. Distracting information often is received when an observer is already engaged in another task (e.g., text messages received while driving a vehicle). As such, in Experiment 3, we manipulated the timing of the onset of the secondary information.

### Methods

Seventy-two new participants were recruited explicitly for Experiment 3 (45 females, *M* age = 18.71). To characterize the extent to which selective attention mechanisms are impaired when information is not time-locked, in Experiment 3 we manipulated the timing of the onset of the HUD-based secondary information (−500, −250, 0, 250, and 500 milliseconds relative to primary visual search task onset). For Experiment 3, we used a mixed factorial design with set size (50 and 80 items) and secondary information onset time as within-subject factors and GG condition (control, glass only, and GG conditions) as a between-subjects factor. All other experimental details were identical to those in Experiment 2.

### Results

**Fig. 4:**
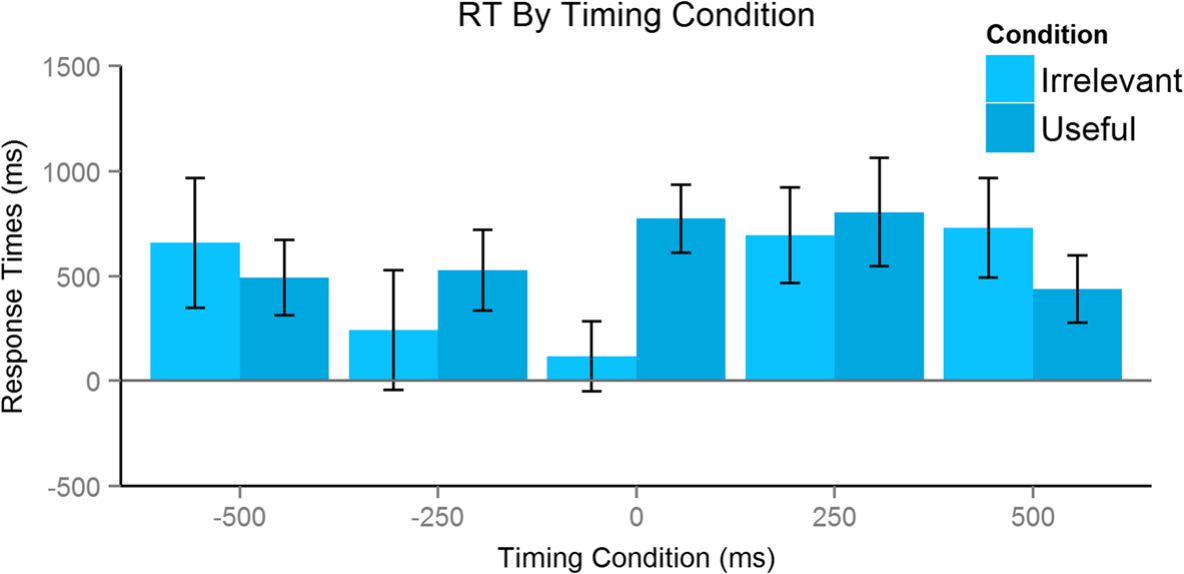
Response times (RTs) relative to control condition (Google Glass condition-control) in Experiment 3 as a function of timing of secondary information onset (relative to primary visual search task) and instruction condition. Error bars indicate SEM

We removed seven participants from the analyses because of accuracy or RT values more than 2 SD from the mean. We found no differences for accuracy across conditions (see Table 1). Because there was no secondary information to manipulate in the control conditions, we conducted two separate analyses of variance: one to evaluate whether there was a cost of secondary information to the primary visual search task compared with the control conditions that collapsed across the timing manipulation (including set size and GG condition), and a second that omitted the control and set size conditions but included the timing manipulation (dual-task GG conditions and temporal onset). For the former, the overall RT data were similar to those derived from Experiments 1 and 2: RT costs were observed when secondary information was presented on the GG (*F*[3, 61] = 3.95, *p* = .013, η^2^ = .160) (see Fig. 2c). Furthermore, planned contrasts indicated that all GG conditions were different from the control conditions (*p*_s_ < .05), but not from each other (*p* = .486). We also found an effect of set size (*F*[1, 61] = 71.46, *p* < .001, η^2^ = .539), but no interaction between set size and GG condition (*F*[3, 61] = 0.89, *p* = .450, η^2^ = .042). RT × set size function slopes averaged 23.25 milliseconds per item. The latter analysis revealed no effect of temporal onset of the secondary information (*F*[4, 120] = 1.24, *p* = .259, η^2^ = .043) or any interaction of secondary information temporal onset with GG condition (*F*[4, 120] = 2.38, *p* = .055, η^2^ = .073) (see Fig. 4). It is perhaps worth noting that visual inspection of Fig. 4 suggests that when the onset of both the primary search task and secondary GG information was concurrent, information that was communicated as irrelevant did not induce a performance cost. This insignificant trend is an outlier to all of our data thus far, that such secondary information produces robust interference with the primary task, and may be related to some change in strategy with regard to attentional deployment associated with the varied-onset timing of the secondary information. Given that the pattern is insignificant and contrary to the first three experiments, as well as being represented by fewer trials than in the previous experiments owing to the additional factor of timing, any conjecture related to it must be made with caution.

Combined, these data suggest that, generally speaking, secondary information induced a cost to the primary visual search task regardless of when it appeared, and they underline how generally distracting HUD-based information may be during multitasking. Again, recognition memory performance was above chance (*p*_s_ < .05), regardless of dual-task condition (*F*[1, 30] = 0.04, *p* = .844, η^2^ = .001).

### Discussion

The results from Experiments 1–3 clearly demonstrate that secondary visual information presented on a HUD interferes with the processing and completion of a concurrent visual task requiring selective attention. It is unclear, however, whether selective attention, which is thought to be serial in nature, represents the bottleneck through which dual-task effects might induce broader performance costs.

## Experiment 4

In our previous experiments, we used a visual search paradigm where the target was difficult to discern from the distractors. In Experiment 4, we altered our primary search task by making the target object red, effectively creating a singleton search task. Importantly, singleton search relies on parallel preattentive mechanisms, as opposed to selective attention (Treisman & Gelade, 1980; Wolfe, 2010). Our goal in Experiment 4 was to evaluate whether the costs associated with irrelevant information presented on the GG are exclusive to tasks in which selective attention mechanisms are required.

### Methods

Seventy-two naive participants were recruited for Experiment 4 (47 females, *M* age = 18.83). All methods were identical to Experiment 2, with one exception. Specifically, we adjusted the color of the target T to red (RGB 237-0-0) to increase saliency and elicit singleton search behavior.

### Results

Ten participants were removed from analyses because of accuracy or RT values more than 2 SD from the mean. We found no differences for accuracy across conditions (see Table 1). RT × set size functions were consistent with patterns reflective of singleton search (average slope of 1.57 milliseconds per item) (Wolfe, 1998). Patterns of RT costs were also similar to those in our previous experiments. There were significant main effects of GG condition (*F*[3, 58] = 9.04, *p* < .001, η^2^ = .319) (see Fig. 2d) and set size (*F*[1, 58] = 13.32, *p* = .001, η^2^ = .187), but no interaction between condition and set size (*F*[3, 58] = 0.63, *p* = .598, η^2^ = .032). We found that the dual-task conditions had slower RTs than the control conditions (*p* < .05). Consistent with Experiments 1–3, performance in the recognition task remained above chance (*p*_s_ < .05) and did not differ across GG conditions (*F*[1, 29] = 0.02, *p* = .883, η^2^ = .001).

### Discussion

These data indicate that interference associated with visual HUD-based distraction is broad, affecting not only selective attention mechanisms but also processes associated with the perceptual extraction of visual features.

## Experiment 5

In Experiments 1–4, the screen on the GG remained blank until a word was presented. As a result, word presentations on the GG could be characterized as abrupt onsets. A large body of literature has shown that such onsets are particularly effective at capturing attentional processes and might be reflexive in nature (Chua, 2013; Folk & Remington, 2015; Theeuwes, Kramer, Hahn, Irwin, & Zelinsky, 1999; Yantis & Jonides, 1984). Given these findings, it is possible that the dual-task costs observed up until this point may not be associated with some limitation in multitasking ability, but rather arose solely from the sudden onset of the secondary stimulus. To test this possibility, in Experiment 5 we presented a persistent visual mask on the GG that was replaced by a word at the onset of the primary visual search task. Finding a pattern of data consistent with Experiments 1–4 would support the assertion that dual-task performance costs associated with HUD-based information are best characterized within the context of basic attentional limitations.

### Methods

Fifty-seven naive participants were recruited for Experiment 5 (26 females, *M* age = 20.11, 19 in each condition). All methods were similar to those in Experiment 2, except that whenever the word was absent from the GG, we presented a visual mask equal in length to the maximum length of the secondary task words (e.g. “#######”). Additionally, given that we found no differences in our previous studies between our two control conditions (i.e., no glass and glass with no words), we included only the no glass control condition.

### Results

**Fig. 5:**
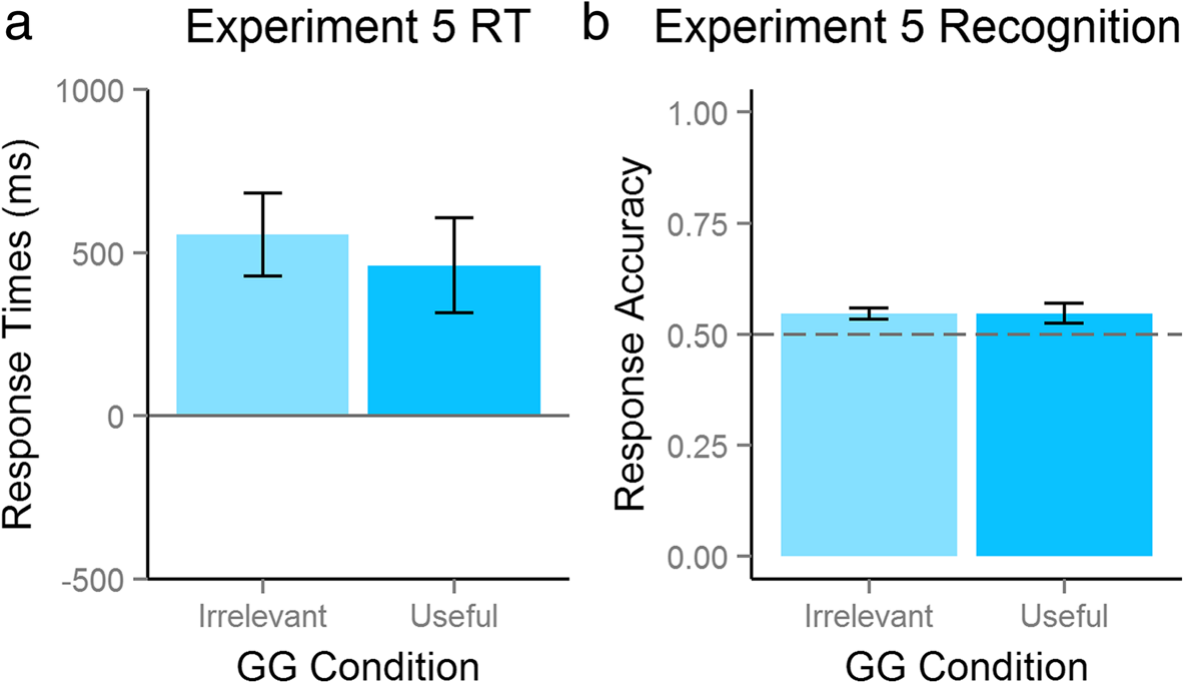
Response times (RTs) relative to control condition (Google Glass [GG] condition-control) in Experiment 5 (**a**) and recognition memory accuracy in Experiment 5 (**b**)

Three participants were removed from the analyses because of accuracy or RT values more than 2 SD from the mean. We found no differences for accuracy across conditions (see Table 1). Patterns of RT costs were also similar to those in our previous experiments (see Fig. 5a). There were significant main effects of GG condition (*F*[2, 51] = 4.89, *p* = .011, η^2^ = .161) and set size (*F*[1, 51] = 75.23, *p* < .001, η^2^ = .596), but no interaction between condition and set size (*F*[2, 51] = 1.37, *p* = .263, η^2^ = .051). We again found that RTs in the dual-task conditions were longer than in the control condition (*p* < .05), regardless of the instructed relevance of the secondary information (*p* = .781). The search slope was 24.86 milliseconds per item. Performance in the recognition task remained above chance (*p*_s_ < .05) and did not differ across GG conditions (*F*[1, 34] = 0.05, *p* = .834, η^2^ = .001) (see Fig. 5b).

### Discussion

Despite the use of a mask to attenuate the abrupt onset of the HUD-based information, the data derived from Experiment 5 were consistent with the patterns observed in Experiments 1–4; participants took more time to complete the primary search task when a secondary stimulus was presented on the GG.

## Conclusions

Overall, our data show that there is a cost associated with wearable technology in dual-task contexts that approximate situations often encountered in the real world. What’s more, this effect is robust and, at the very least, difficult to mitigate. In Experiment 1, we found evidence of a dual-task cost when wearing the GG and that that cost was not offset by relevance instructions pertaining to the secondary information; costs persisted even when participants were instructed to ignore the secondary information. In Experiment 2, we found that when participants were informed that they would be tested on the secondary information, performance costs were even more robust. In Experiment 3, we showed that RT costs associated with the HUD-based secondary information were largely orthogonal to the temporal onset of that information in relation to the primary task; secondary information was nearly always disruptive to visual search, regardless of time of onset. Experiment 4 indicated that the costs of secondary HUD-based information are not only incurred to selective attention mechanisms, but are in fact present at early processing stages thought to be associated with preattentive mechanisms. Finally, in Experiment 5, we tested the possibility that the patterns of data observed in Experiments 1–4 may have been associated with the abrupt onset of the HUD-based information and found that the pattern persisted when the abrupt onsets were eliminated.

Our results provide robust evidence that primary task performance is impaired by secondary information presented on a wearable HUD and is relatively independent of task relevance. Although there was some evidence that participants weighed secondary information portrayed as relevant to the primary task in Experiment 2 more heavily than information portrayed as irrelevant, and in turn had larger overall performance costs, this finding was not replicated in all experiments. Generally speaking, information pertinence may not matter when set against broader distraction, as previous researchers have found that items relevant to safety were not recognized any more often than irrelevant items in either single- or dual-task scenarios (Strayer & Drews, 2007). That these costs exist in a simplified environment is particularly worrisome when speculating about how they might generalize to more realistic multitasking situations (Horrey & Wickens, 2006). Even under relatively simple task conditions, performance decrements were substantial, at ranges of 450–600 milliseconds compared with control conditions. Given the practicality and growing practice of implementing HUDs for a wider variety of users (beyond those in aviation), these costs should give researchers and practitioners pause (Crawford & Neal, 2006; Liu et al., 2009). It is not unreasonable to speculate that these costs might be more severe under increasingly complex, realistic task conditions (e.g., when driving) (Strayer et al., 2003). In simulated environments, GG has produced impairments similar to those present when using a cellular device; however, the performance decrements are less severe (He, Choi, McCarley, Chaparro, & Wang, 2015; Sawyer, Finomore, Calvo, & Hancock, 2014).

Importantly, in our studies, performance impairments were present regardless of whether the primary task depended on preattentive parallel processes or serial attention, suggesting that costs under real-world conditions are likely to occur across a broad array of tasks and conditions. Whereas previous findings have demonstrated impairments in perceptual memory under dual-task conditions (Strayer et al., 2003), our data suggest broad-spectrum impairments to attentional processes as well. Our findings are consistent with those derived from previous theoretical models suggesting that cross-task interference is likely to be high when competing information is presented within the same perceptual modality (Wickens, 2008). However, the cognitive mechanisms underlying the interference are often left unspecified. Strayer and Drews (2007) proposed that the underlying interference accompanying technology-based distraction is likely associated with inattentional blindness; secondary information impedes the encoding of primary task information. Our finding that secondary information can impede processes associated with both inefficient and efficient search suggests that dual-task performance impairments may actually arise quite early in the information-processing chain and impact the selection of which low-level information in the environment is passed on to higher-order processes for scrutiny. This explanation is not inconsistent with the proposal of Strayer and Drews. Rather, it provides some broader perspective on where their inattentional blindness findings may emerge from: broad impairments to the deployment of attentional processes. Still, it is worth noting that while we found evidence for impairments to both parallel and serial attentional mechanisms through our experimental manipulations, within our studies there was no interaction of GG condition with set size in the primary visual search task, which one might expect to observe in the presence of selective attention impairments (though this might also be reflective of some sort of decision-making process as opposed to selective attention alone). This might suggest that the locus of dual-task impairments, at least as it pertains to our particular set of tasks, is more complex than can be described by attentional impairments alone. In future work, researchers should continue to explore the phenomena at the mechanistic level.

Overall, performance costs in our studies occurred regardless of perceived importance of secondary information (participants were unable to ignore secondary information even when instructed to do so) and time course of information presentation. Combined, our data strongly suggest that caution should be exercised when deploying HUD-based informational displays in circumstances where the primary user task is visual in nature. Just because we can, does not mean we should.
